# Polyethyleneimine Capped Silver Nanoclusters as Efficient Antibacterial Agents

**DOI:** 10.3390/ijerph13030334

**Published:** 2016-03-18

**Authors:** Dong Xu, Qingyun Wang, Tao Yang, Jianzhong Cao, Qinlu Lin, Zhiqin Yuan, Le Li

**Affiliations:** 1 National Engineering Laboratory for Rice and By-Products Further Processing, Central South University of Forestry & Technology, Changsha 410004, China; jalywang6688@163.com (Q.W.); yangtao807@163.com (T.Y.); cjz2007@hotmail.com (J.C.); 2 State Key Laboratory of Chemical Resource Engineering, Beijing University of Chemical Technology, Beijing 100029, China; yuanzq@mail.buct.edu.cn; 3 Hunan Ehome Health Technology Inc., Buliding 6# 1715, LvDi Central Square, Yuelu District, Changsha 410023, China; simonli111@hotmail.com

**Keywords:** silver nanoclusters, polyethyleneimine, antibacterial agent

## Abstract

Development of efficient antibacterial agents is critical for human health. In the present study, we investigated the antibacterial activity of polyethyleneimine (PEI)-capped silver nanoclusters (PEI-AgNCs), based on the fact that nanoclusters normally have higher surface-to-volume ratios than traditional nanomaterials and PEI itself has a strong antimicrobial capacity. We synthesized stable silver nanoclusters by altering PEI molecular weight from 0.6 kDa to 25 kDa and characterized them by UV-Vis absorption and fluorescence spectroscopy and high resolution transmission electron microscopy. The sizes of AgNCs were around 2 nm in diameter and were little influenced by the molecular weight of PEIs. The antibacterial abilities of the four PEI-AgNCs were explored on agar plate and in liquid systems. Our results revealed that the antibacterial activity of PEI-AgNCs is excellent and the reduction of PEI molecular weight could result in the increased antibacterial capacity of PEI-AgNCs. Such proposed new materials might be useful as efficient antibacterial agents in practical clinical applications.

## 1. Introduction

Due to the overuse of antibiotics in human patients and livestock, antibiotic-resistant infections are increasingly emerging as a serious threat to human health care [1,2,3]. On the other hand, microbial contamination in food processing and packing, the textile industry [4], water purification [5] and medical devices [6,7] is widespread Therefore, developing high activity and broad spectrum antibacterial agents is of great importance to prevent the threat from pathogens with antibiotic resistance. Toward this goal, various materials with high toxicity have been exploited as antibacterial agents, such as TiO_2_, CuO and quaternary phosphonium compounds [8,9]. Although these antibacterial agents show high antibacterial activity, some drawbacks like low stability and complicated preparation processes limit their practical application. Thereby, exploiting stable antibacterial agents through simple routes is still appealing.

Silver materials have been proved to exhibit powerful antibacterial ability during the last two decades [10]. Among those materials, silver nanoparticles (AgNPs) have attracted wide research interest in the past few years due to their strong antibacterial ability and broad inhibitory spectra for different microorganisms and low toxicity to mammalian cells [11,12]. AgNPs could cause antimicrobial effects through various approaches [13,14]. For example, silver ion can be released by AgNPs and these ions inactivate many crucial enzymes through their interaction with thiol groups, resulting in the inhibition of cell functions and the cell death. AgNPs also have the ability to anchor to the cell wall of bacteria and penetrate it, leading to the disruption of respiration and incresed membrane permeability. The generation of free radicals induced by AgNPs under light is another way to cause bacterial death. However, the preparation of a uniform and stable colloidal dispersion of AgNPs without agglomeration or precipitation is still a problem to be solved prior to any practical application. On the other hand, several reports [15,16] have described that the antibacterial properties of AgNPs are size-dependent and smaller AgNPs showed greater antimicrobial activity by increasing the release rate of silver ion and the contact surface area with bacteria. Moreover, as a new kind nanomaterial, silver nanoclusters (AgNCs) that consist of several to hundreds of atoms and are less than 2 nm in size have attracted great attention due to their physicochemical properties [17,18]. Despite their wide applications in biomedical fields such as cell imaging, sensing, solar cells and photodynamic therapy, *etc*. [19], studies of the antibacterial ability of AgNCs are still limited. 

Polyethylenimines (PEIs) are synthetic polymers containing primary, secondary and tertiary amine groups with the ratio of 1:2:1. They are highly basic and positively charged, having been extensively used as a vehicle for non-viral gene delivery and therapy [20]. In the field of microbiology, PEIs not only can enhance the bactericidal efficiency of both hydrophilic and hydrophobic antibiotics [21], but also is a common microbicidal ingredient by itself. They have permeabilizing effects and can disrupt bacterial cell membranes [22,23]. PEIs incorporated into composite resins [24] or provisional cements [25] demonstrated a stable and long-lasting antibacterial effect. Furthermore, PEIs were employed as ligands for preparing or modifying metal nanomaterials due to their strong metal-amine interactions, and the positively charged PEI ligands can largely prevent nanomaterial aggregation [26,27]. Utilizing PEI as template and stabilizer, AgNCs have been readily synthesized and applied to sense halide ion [28], copper ion [29], pH value [30], Sudan I–IV [31] and glucose [32]. However, studies on the antibacterial properties of PEI-capped AgNCs (PEI-AgNCs) are still very rare. In fact, PEI-AgNCs should possess high antibacterial efficiency because both the PEI ligand and AgNCs have been reported to exhibit antibacterial activities. This fact provoked our interest in exploring the antibacterial activity of PEI-AgNCs. 

In this article, the PEI-capped AgNCs in a stable dispersion were prepared in room temperature for antibacterial applications in the presence of ascorbic acid and PEI. A series of PEI-AgNCs were synthesized by altering PEI molecular weight from 0.6 kDa to 25 kDa. By utilizing *Escherichia coli* (*E. coli*) as a model, PEI-AgNCs have been proven to possess unusual antibacterial activity. PEI-AgNCs made from smaller PEIs are particularly suited for bactericidal applications.

## 2. Materials and Methods 

### 2.1. Chemicals

Branched polyethyleneimines (MW 0.6k, 1.8k, 10k, and 25k) were bought from Alfa Aesar (Heysham, UK). Ascorbic acid, silver nitrate (AgNO_3_), sodium hydroxide (NaOH), sodium borohydride (NaBH_4_), sodium chloride (NaCl), polyvinylpyrrolidone (PVP MW 25k), acetic acid (HAc) and other chemicals were purchased from Sinopharm Chemica Reagent Corporation (Shanghai, China). *E. coli* (ATCC 10536) was obtained from the American Type Culture Collection (Manassas, VA, USA). Tryptone and yeast extract used for Luria Bertani (LB) medium preparation were bought from Oxoid Ltd. (Basingstoke, UK). 

### 2.2. Syntheses of PEI-AgNCs and AgNPs 

In a typical procedure [29], 25 μL of 0.1 M AgNO_3_ was added to 10 mL 0.625% (wt) PEI stock solution and the solution was continually stirred for 2 h to ensure thorough complexation between Ag^+^ and the amine ligands. Then, the solution pH was adjusted to 5 with HAc solution. After 30 μL of 0.1 M AA was added to the solution, the continuous stirring was kept for 2 days. Altering the PEI molecular weights from 0.6 kDa to 25 kDa under same mass concentration conditions would produce PEI0.6k-AgNCs, PEI1.8k-AgNCs, PEI10k-AgNCs and PEI25k-AgNCs, respectively. AgNPs were also prepared according to the previous literature with slight modifications [33]. Briefly, 25 μL of 0.1 M AgNO_3_ was dripped to 10 mL solution containing 0.008 M NaBH_4_ while stirring in an ice bath at approximately one drop per second. After AgNO_3_ is added, 100 μL of 6% PVP was injected into solution to stabilize the AgNPs. These two nanomaterials were characterized systematically. The UV–Visible absorption spectra of PEI-AgNCs and AgNPs were obtained with a UV-1800 spectrophotometer (Suzhou Shimadzu Instrument Co., Ltd., Suzhou, China). The fluorescence spectra were measured using a F-2700 fluorescence spectrophotometer (Hitachi, Tokyo, Japan). High resolution transmission electron microscopy (HRTEM) images were collected with a H-7500 high resolution transmission electron microscope (Hitachi).

### 2.3. Antibacterial Tests 

A standard disk diffusion method [34] was employed to evaluate the antimicrobial properties of the various PEI-AgNCs. The as-prepared AgNPs, chloramphenicol and different PEI solutions were also tested for comparison. The discs impregnated with PEI-AgNCs, PEIs, AgNPs, chloramphenicol and distilled water were obtained by loading the filter papers (7 mm in diameter) with 20 μL solutions. In particular, all of PEI-AgNCs contained 62.5 mg/mL PEI of different molecular weights and 27 μg/mL silver. The concentrations of all PEI suspension were 62.5 mg/mL. The silver concentration of AgNPs solution was 27 μg/mL. The mass fraction of chloramphenicol as positive control was 0.01%. 10^8^ cfu/mL *E. coli* suspensions in LB liquid medium were incubated on agar plates in aseptic environment. All discs were placed on the top of agar one by one carefully. Then, the plates were kept at 37 °C for 24 h. After incubation, the photos of plates were taken by Iphone4 mobile phone and then were further processed by ImageJ software.

Minimum inhibitory concentrations (MICs) were obtained by means of serial twofold dilution methods according to the Clinical and Laboratory Standards Institute [34]. Twofold serial dilutions of tested solutions were prepared in tubes by using sterile distilled water with PEI concentration from 31.25 mg/mL to 0.24 mg/mL and silver concentration from 13.50 μg/mL to 0.11 μg/mL. One hundred microliter of these solutions was transferred into 96-well microliter plates in sequence. Next, 100 μL of the prepared *E. coli* suspensions with 10^5^ cfu/mL were injected into each well. The lowest concentration that that leaded to no microbial growth was determined to be MIC. To further confirm the MIC of these materials against *E. coli*, we mixed 1 mL the solution at MIC with 1 mL 10^3^ cfu/mL *E. coli* suspensions for 3 min. Then 1 mL of the mixture was drawn and injected to the culture dish with a diameter of 10 cm. Finally, 15 mL of LB nutrient agar at 46 °C in wash bath was added to each culture dish and the dishes were rotated to homogenize the liquid. The procedures were repeated by using sterile water as control. After the solidification of agar, the dishes would be kept at 37 °C for 24 h and then imaged. 

The inhibition ratio based on the bacterial optical density was also compared between PEI-AgNCs and other materials. Briefly, 10^8^ cfu/mL *E. coli* suspensions with optical density value at 0.5 was diluted 1000 times by broth at first. Then, 200 μL of the tested solutions including PEI-AgNCs (PEI, 62.5 mg/mL + silver, 27 μg/mL), PEI (62.5 mg/mL), silver nanoparticle colloids (27 μg/mL) and distilled water was diluted to 1 mL with sterilized physiological saline and then 1 mL prepared bacteria suspensions were added. After incubation at 37 °C for 24 h, the absorbance of the bacterial broth medium at 650 nm was measured by a UV-Vis spectrophotometer. The inhibition ratios were calculated through the relative absorption intensity:

R = 100−100 (I_s_−I_0_)/(I_water_−I_0_) (1)

where, R is inhibition ratio; I_s_ and I_water_ are the absorption intensity of bacterial suspension at 650 nm with samples and water after incubation; and I_0_ is the absorption intensity of mixed solution composed of 1 mL physiological saline and 1 mL prepared bacteria suspensions at 650 nm before incubation. 

## 3. Results

### Preparation of PEI-AgNCs and AgNPs and Their Antibacterial Properties.

#### Syntheses and Characterization of PEI- AgNCs and AgNPs

**Figure 1:**
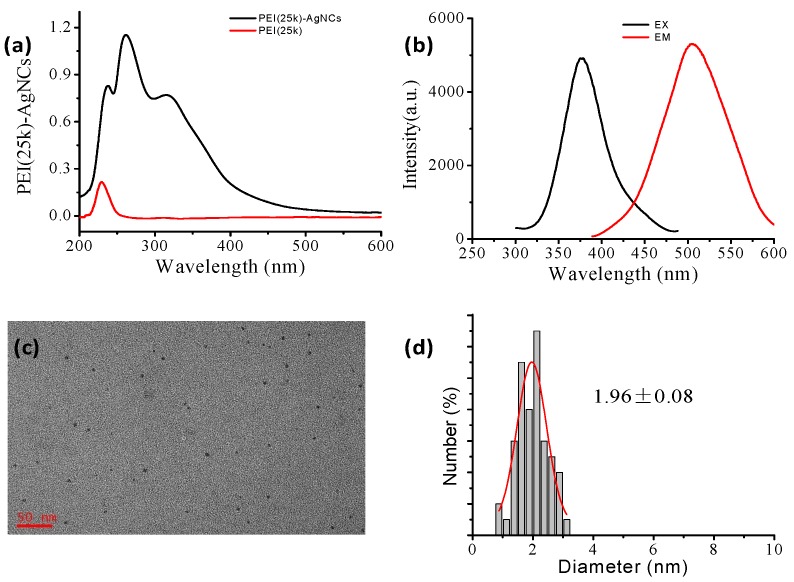
Characterizations of PEI25k-AgNCs. (**a**) The UV-Vis spectra of PEI-AgNCs (dark curve) and PEI (red curve); (**b**) The excitation (dark curve) and emission (red curve) spectra, where EX/EM represents the excitation/emission wavelength; (**c**) Their HRTEM image; (**d**) The corresponding statistic diameter.

The detailed preparation procedure for synthesizing PEI capped AgNCs by using PEI25k was according to the earlier literature with slight modification [29], and they were characterized by UV-Vis spectrometry, steady-state fluorescence spectrometry and HRTEM. The UV-Vis spectra of PEI25k and PEI25k-AgNCs are provided in Figure 1a. Three obvious absorption peaks of PEI-AgNC solution centered at 229 nm, 260 nm and 315 nm were observed, respectively. Among them, only the absorption band of 315 nm is attributed to AgNCs, while the bands around 229 nm and 260 nm are originated from the PEI and the oxidized PEI [35], respectively. This fact is also proved by the UV-Vis spectrum of sole PEI solution. An important feature of silver nanoclusters is that they can be regarded as a class of fluorophores with high brightness and photostability. Therefore, in order to verify the obtained materials are AgNCs, we measured their fluorescence spectra, see Figure 1b. The excitation and emission spectra of the solution are similar to that reported previously [29], where the excitation peak of PEI25k-AgNCs is located at 378 nm and the emission peak is around 508 nm. The HRTEM images of PEI25k-AgNCs display their average diameter is 1.96 nm, as is illustrated in Figure 1c,d. These results demonstrated PEI25k-AgNCs were successfully prepared.

**Figure 2:**
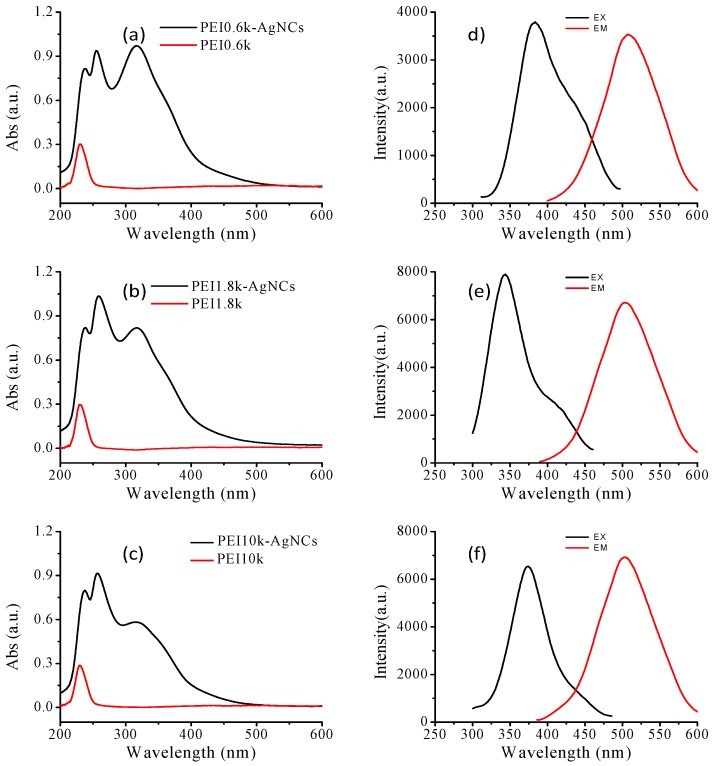
Characterizations of PEI-AgNCs and PEIs with PEI molecular weight from 0.6 kDa to 10 kDa. (**a**–**c**) The UV-Vis spectra of PEI-AgNCs and PEIs with PEI molecular weight of 0.6 kDa (**a**); 1.8 kDa (**b**) and 10 kDa (**c**). (**d**–**f**) The excitation and emmision spectra of PEI0.6k-AgNCs (**d**); PEI1.8k-AgNCs (**e**) and PEI10k-AgNCs (**f**), respectively.

Since PEIs with different molecular weights showed various antibacterial activity [36], we also prepared PEI-AgNCs by utilizing PEI0.6k, PEI1.8k and PEI10k as the capping ligands. The UV-Vis spectra, excitation and emission spectra and the sizes of these materials are provided in Figure 2 and Supporting Figure S1. A nearly fixed UV-Vis absorption peak at 315 nm was observed in all AgNCs prepared with different PEIs. The maximum excitation fluorescence peaks of PEI-AgNCs, however, are slight different. For example, the maximum excitation wavelengths of PEI0.6k-AgNCs, PEI1.8k-AgNCs and PEI10k-AgNCs are located at 383 nm, 346 nm and 376 nm, respectively. Despite of the slight difference in excitation spectra, all obtained PEI-AgNCs show similar emission maxima that are around 506 nm. However, the HRTEM images demonstrated the diameters of PEI0.6k-AgNCs, PEI1.8k-AgNCs and PEI10k-AgNCs are all approaching to 2.0 nm. Take together, we can see the optical properties of PEI-AgNCs were little influenced by PEI molecules used for preparation.

**Figure 3:**
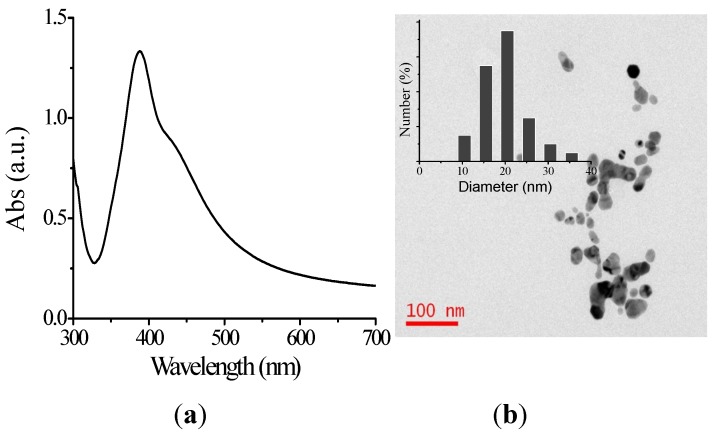
The UV-Vis absorption spectrum (**a**) and the HRTEM image (**b**) of AgNPs. The inset is their statistic diameters.

In addition, AgNPs were also produced for antibacterial investigation as a comparison, see Figure 3a,b. After the addition of silver nitrate, the solution turned bright yellow from colorless, suggesting the formation of AgNPs. The surface plasmon resonance peak of AgNP solution is at 390 nm. The diameter of AgNPs was determined to be 21.2 ± 1.3 nm by HRTEM (100 counts).

#### The Assessment of PEI-AgNCs Antibacterial Activity

Silver materials and PEIs have been recognized as efficient biocides against a broad-spectrum bacteria [13,14,22,23]. Focusing on the prospective application of the as-prepared PEI-AgNCs as the antibacterial materials, *E. coli* was selected as a model to evaluate their antibacterial abilities both on agar plates and in liquid systems. 

**Figure 4:**
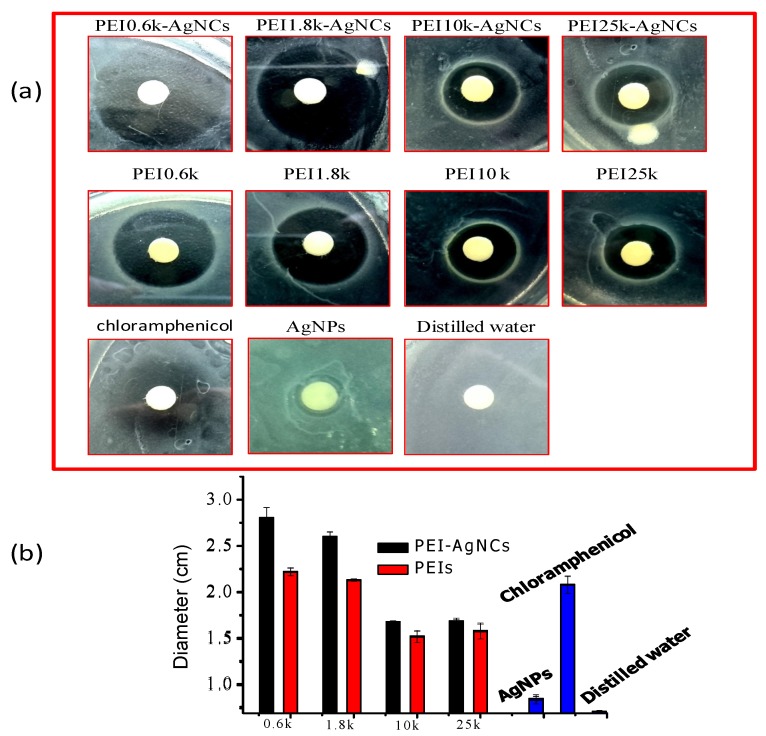
The inhibition zones yielded by PEI-AgNCs. PEIs, AgNPs and the positive and negative controls for *E. coli*. (**a**) Their images. The size of each image is 3 cm × 3 cm; (**b**) The statistic results of the diameters of these zones.

The representative images of inhibition zones yielded by PEI-AgNCs, PEIs, AgNPs and the positive and negative controls are shown in Figure 4a, whose statistical diameters are also shown in Figure 4b. The inhibition zones of PEIs and PEI-AgNCs are obvious and the reduction of PEI molecular weight from 25 kDa to 0.6 kDa can enhance the antibacterial abilities of both PEIs and PEI-AgNCs. The antibacterial effect of PEI-AgNCs, however, are always better than that of the corresponding PEI because of the presence of silver although its content is low. For example, PEI0.6k-AgNCs were found to be the best antibacterial agent in our study and the diameter of the inhibition zone by PEI0.6k-AgNCs was 0.6 cm larger than that of PEI0.6k. In PEI-AgNCs produced by larger PEI molecules, the antibacterial role of silver could be partially inhibited by steric hindrance from PEI. In comparison, 21.2 nm AgNPs only showed slight antibacterial zone near the disc. Furthermore, it should be noticed that the inhibition zones of PEI0.6k-AgNCs and PEI1.8K-AgNCs are even larger than that of 0.01% chloramphenicol, indicating PEI-AgNCs might be an alternative antibacterial agent to uneasy degradation chloramphenicol.

**Figure 5:**
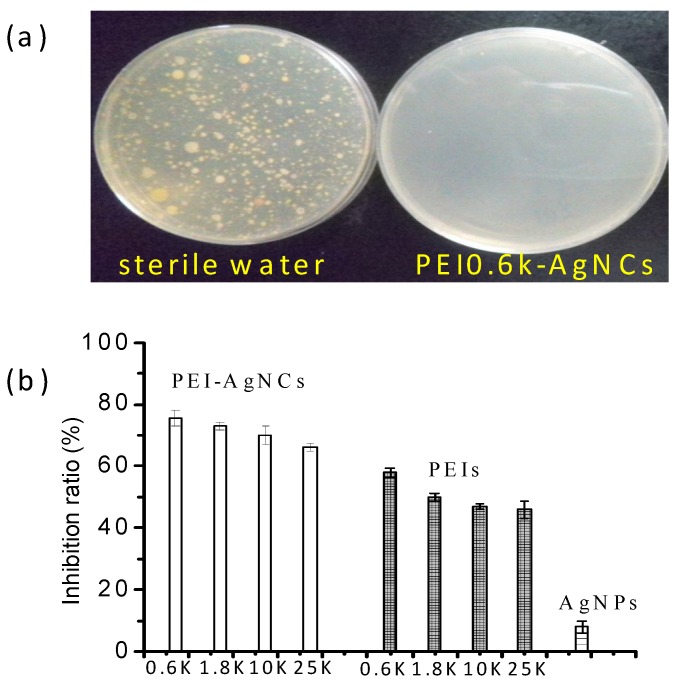
The strong antibacterial activities of PEI-AgNCs. (**a**) Photographs of *E. coli* colonies on broth agar plates in the presence of sterile water and 7.81 mg/mL PEI0.6k-AgNCs. It seems that the sterile water or *E. coli* suspension used for the experiment was contaminated by other bacteria, but it has no effect on verifying the MICs of PEI-AgNCs; (**b**) The inhibition ratios of PEI-AgNCs, PEIs and AgNPs, which were calculated according to Equation 1.

The MIC, defined as the lowest concentration of material that inhibits the growth of an organism [37], was obtained based on batch cultures containing varying concentrations of antibacterial ingredients. A series of *E. coli* suspension were prepared in the LB liquid medium and cultured for 24 h in the presence of PEI-AgNCs, PEIs, AgNPs and sterilized physiological saline, respectively. We found that the bacterial suspensions with sterilized physiological saline become turbid after incubation, illustrating *E. coli* proliferated rapidly. The addition of PEI0.6k-AgNC below 7.81 mg/mL could not efficiently retard the growth of *E. coli* and the mixture solution still turned turbid after 24 h incubation, while PEI0.6k-AgNC higher than 7.81 mg/mL caused the solution to become pellucid. The absence of obvious absorbance at 650 nm under UV-Vis spectroscopy further confirmed that the MIC of PEI0.6k-AgNCs was 7.81 mg/mL. Actually, at MIC, no bacterial colony was found on nutrient agar plate (Figure 5a). Similarly, The MIC of both PEI1.8k-AgNCs and PEI0.6k was determined to be 15.63 mg/mL, while the MICs of other PEI-AgNCs and PEIs are higher than 15.63 mg/mL. Based on the results, we can conclude that silver indeed played a more important role in antibacterial behaviors in PEI-AgNCs complexes made with PEI of lower molecular weight. The MIC of AgNPs can not be achieved because *E. coli* still proliferated fast at the concentration 13.50 μg/mL. We also intended to obtain the MIC of the mixture of PEIs and AgNPs. But the aggregation of negatively charged AgNPs in the presence of positively charged PEI hindered the determination. 

The inhibition ratios of PEI-AgNCs, PEIs and AgNPs are shown in Figure 5b, in which the ultimate concentration of PEIs is 6.25 mg/mL and silver concentration is 2.7 μg/mL. Obviously, PEI-AgNCs exhibit strong antibacterial activity. The inhibition ratios of four PEI-AgNCs are higher than 75%, while the ratios of PEIs are nearly 50%. Again, the reduction of PEI molecular weights could increase the antibacterial properties of both PEIs and PEI-AgNCs. AgNPs still showed antibacterial activity at low concentration in solution probably because AgNPs can diffuse freely in solution and the chance of interaction between AgNPs and *E. coli* in solution was increased. 

In short, PEI-AgNCs showed excellent antibacterial properties. Their bactericide effects were enhanced by decreasing PEI molecular weight.

## 4. Discussion

### 4.1. The Formation of PEI-AgNCs 

PEIs play a key role in metal nanoparticle fabrication because they have abundant amine motifs that can form stable complexes with metal atoms via strong coordination. After the absorption of large polymer PEIs onto a nanoparticle surface, steric effects would make them very stable and dispersed. Therefore, monodisperse gold nanoparticles [38], AgNPs [39] and quantum dots [40] were already synthesized in the presence of PEIs. Similarly, PEIs could provide scaffolds for AgNCs preparation. We prepared PEI25k-AgNCs according to the previous study [29]. Adjusting the PEI solution pH value to 5 is important for AgNCs formation. After the reduction by asorbic acid, we presumed large AgNPs were not formed because no characteristic surface plasmon band was observed in the range of 400–500 nm [41]. Further HRTEM results indicated the sizes of PEI25k-AgNCs are nearly the same as those reported [28]. The obtained PEI-AgNCs were very stable during long-time storage due to PEI capping, which facilitated their practical applications. 

PEIs have been used in composite resins and provisional cements for their prominent antibacterial abilities [24,25]. Also, some reports demonstrated that the molecular weight of PEIs affects their antibacterial abilities [36]. Hence, it is necessary to prepare PEI-AgNCs for antibacterial study by altering the PEI molecular weight. We found that the molecular weight of PEIs showed little influence on the UV-Vis absorption spectra, the emission spectra and the sizes of PEI-AgNCs. While a phenomenon has been observed that the excitation maxima of PEI-AgNCs irregularly varied under different PEI molecular weights.

The surface-to-volume ratio of nanoparticles exhibit an important role in bacteria inhibition, which is closely related to the diameter, so decreasing the diameter could enhance the ratio effectively. In contrast to PVP-capped AgNPs with 21.2 nm in diameter, the surface-to-volume ratio of AgNCs of the same mass could be increased by 1.37 × 10^4^ times. Consequently, AgNCs are more suitable for antibacterial applications. 

In summary, we fabricated PEI-AgNCs at room temperature with different PEIs. The produced AgNCs have some superior properties, including long-time dispersion stability, large surface-to-volume ratio and high antibacterial activity. The sizes of AgNCs were not affected by changing the PEI molecular weights.

### 4.2. Antibacterial Properties of PEI-AgNCs 

The results implied that the antibacterial abilities of PEI-AgNCs mainly stem from PEI. This is rational because the amount of PEI for AgNC preparation is huge and PEIs themselves display strong antibacterial properties. Nevertheless, the function of silver cannot be neglected. Silver indeed made contributions to the antibacterial property of PEI-AgNCs as the antibacterial effects of PEI-AgNCs are more obvious than that of the corresponding PEIs. Notice that the silver amount used in present study is optimal for prepare high-fluorescence AgNCs, which may not represent the best antibacterial abilities of PEI-AgNCs. Other studies should be further done to investigate the influence of silver content on the antibacterial ability of PEI-AgNCs. 

There is an obvious feature in our results that antibacterial ability of PEI-AgNCs is affected by the molecular weight of the PEI used for AgNC synthesis in the range of 0.6 kDa–25 kDa. We speculate several reasons may account for this. Utilizing small PEI molecules in the preparation would result in PEI-AgNCs complexes with small diameter, which would diffuse faster. As a result, PEI0.6k-AgNCs have more opportunities to collide with bacteria in contrast to other complexes. Generally, AgNCs were supposed to be enwrapped by PEI molecules. Small molecular PEI could provide small steric hindrance. Thereby, silver was more likely to be exposed outside to contact with bacterial surface and kill microorganisms. The released silver ions can reach bacteria over a short distance and with high concentration. Although the antibacterial activities of PEI-AgNCs produced by large PEI molecules have weak antibacterial activities of AgNCs, we believed their antibacterial effects would last longer. Nevertheless, PEI-AgNCs synthesized with small PEI molecules are commercially applicable as antibacterials without the deffects of antibiotics to the human body. 

We suppose that the antibacterial mechanisms of PEI-AgNCs are very complex, combining the advantages of PEI and silver. PEI-AgNCs could readily be absorbed and enriched on negatively charged bacterium surfaces due to the positive charge of PEI. PEI could still cause permeabilizing effects and disrupt bacteria. Through the interaction, silver is carried to cell surface and its antibacterial activity would be apparently enhanced. Silver ions released from AgNCs could have a high concentration surrounding bacteria and kill them rapidly. Also, AgNCs can function through breaking the permeability of the outer membrane, inhibiting bacterial respiration and growth, and destroying the membrane structure.

## 5. Conclusions 

We successfully synthesized various stable PEI-AgNCs and investigated their antibacterial activities. It was found that changing the PEI molecules did not affect the size of AgNCs and the PEI-AgNCs possessed high antibacterial ability. The reduction of PEI molecular weight could provide PEI-AgNCs with higher antibacterial properties. PEI-AgNCs have great application value in the field of antibacterial agents. 

## Supplementary Materials

The following are available online at http://www.mdpi.com/1660-4601/13/3/334/s1, Figure S1: The HRTEM images and the corresponding statistic diameters of PEI-AgNCs with PEI molecular weights from 0.6 kDa–1.8 kDa.

## Abbreviations

AgNC	Silver nanocluster
AgNPs	Silver nanoparticles
HRTEM	High resolution transmission electron microscopy
LB	Luria Bertani
MICs	Minimum inhibitory concentrations
PEI	Polyethyleneimine
PEI-AgNCs	Polyethyleneimine capped silver nanoclusters
PVP	Polyvinylpyrrolidone
EX/EM	Excitation/emission wavelength
