# Supplementary material for: Polyethyleneimine Capped Silver Nanoclusters as Efficient Antibacterial Agents
Source: Int J Environ Res Public Health. 2016 Mar 18;13(3):334. doi: 10.3390/ijerph13030334 (PMC4808997; doi:10.3390/ijerph13030334)
Supplement: Supplementary file 1 [file ijerph-13-00334-s001.pdf]

Supplementary Materials: Polyethyleneimine Capped Silver Nanoclusters as Efficient Antibacterial Agents

Dong Xu, Qingyun Wang, Tao Yang, Jianzhong Cao, Qinlu Lin, Zhiqin Yuan and Le Li

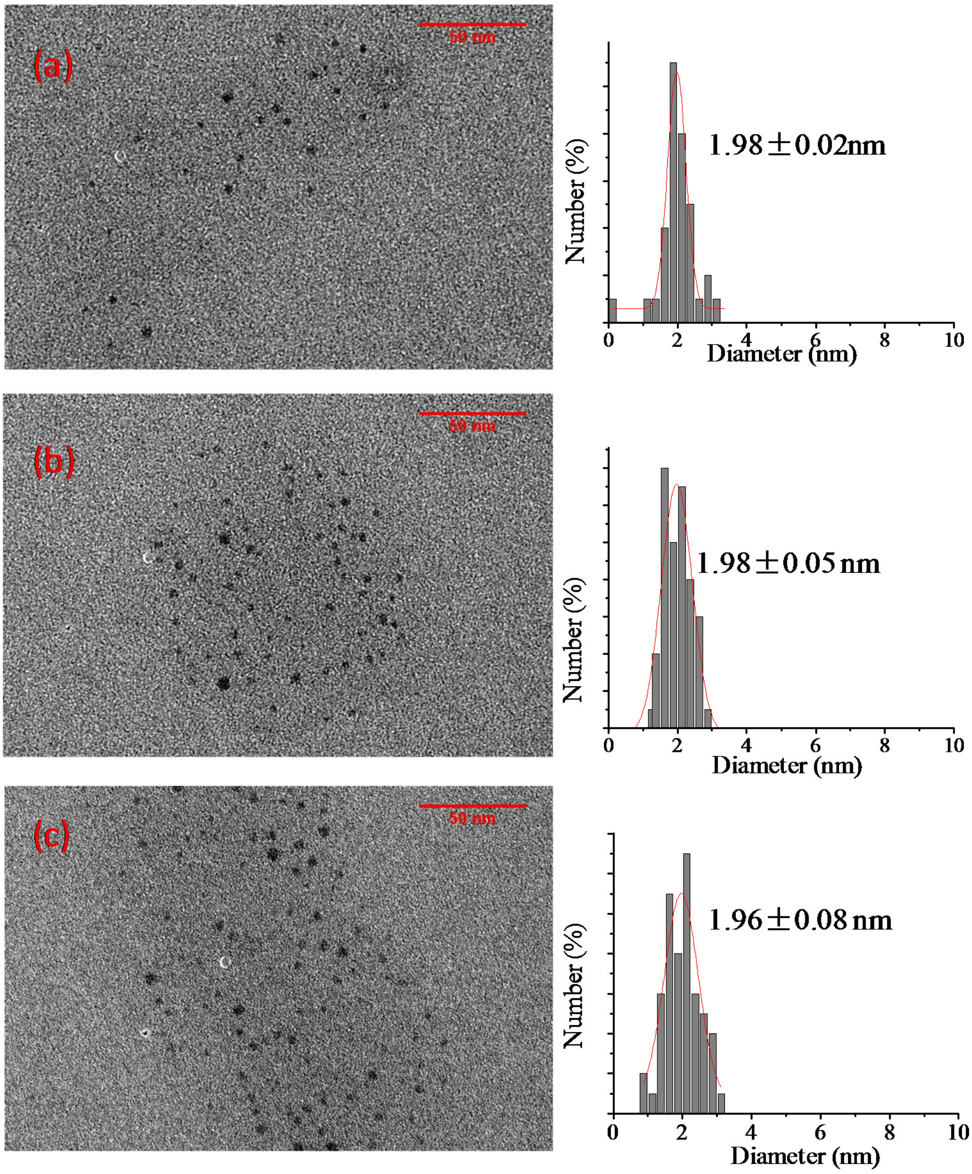

Figure S1: The HRTEM images and the corresponding statistic diameters of PEI0.6k-AgNCs (**a**); PEI1.8k-AgNCs (**b**) and PEI10-AgNCs (**c**).

© 2016 by the authors; licensee MDPI, Basel, Switzerland. This article is an open access article distributed under the terms and conditions of the Creative Commons by Attribution (CC-BY) license (http://creativecommons.org/licenses/by/4.0/).
